# Vascular Endothelial Growth Factor +936C/T, –634G/C, –2578C/A, and –1154G/A Polymorphisms with Risk of Preeclampsia: A Meta-Analysis

**DOI:** 10.1371/journal.pone.0078173

**Published:** 2013-11-04

**Authors:** Daye Cheng, Yiwen Hao, Wenling Zhou, Yiran Ma

**Affiliations:** Department of Transfusion, The First Hospital of China Medical University, Shenyang, China; Gentofte University Hospital, Denmark

## Abstract

**Background:**

Emerging evidence showed that VEGF gene polymorphisms are involved in the regulation of VEGF protein expression, thus increasing an individual's susceptibility to preeclampsia (PE); but individually published results are inconclusive. The aim of this meta-analysis was to investigate the associations between VEGF gene polymorphisms and PE risk.

**Methods:**

A systematic literature search of MEDLINE, Embase, Web of Science, and CNKI (Chinese National Knowledge Infrastructure) databases was conducted. Statistical analyses were performed using STATA 12.0 software and Review manager 5.1. Odds ratios (ORs) with 95% confidence intervals (CIs) were used to assess the strength of associations.

**Results:**

According to the inclusion criteria, 11 case-control studies were finally included in this meta-analysis. A total of 1,069 PE cases and 1,315 controls were included in this study. Our meta-analysis indicated that VEGF +936C/T (T vs. C, OR = 1.52, 95%CI = 1.08–2.12) or −634G/C polymorphism (C vs. G, OR = 1.24, 95% CI = 1.03–1.50) was associated with the risk of PE, whereas there was no association between −2578C/A (A vs. C, OR = 0.98, 95%CI = 0.82–1.16) or −1154G/A (A vs. G, OR = 1.30, 95%CI = 0.94–1.78) polymorphism and PE risk in our study.

**Conclusion:**

Our meta-analysis suggested that VEGF −2578C/A or −1154G/A polymorphism had no association with PE risk in all examined patients, whereas there was an association between VEGF +936C/T or −634G/C polymorphism and risk of PE.

## Introduction

Preeclampsia (PE), a human-pregnancy-specific disease defined as the occurrence of hypertension and significant proteinuria in a previously healthy woman on or after the 20th week of gestation, occurs in about 2–8% of pregnancies [1]–[3]. Despite its prevalence and severity, the pathophysiology of PE is still not completely understood. PE is now thought to result from a combination of immunologic, inflammatory, dietary, and genetic factors that lead to the failure of normal trophoblastic invasion and remodeling of the uterine spiral arteries [4]. Therefore, the role of the imbalance between angiogenic and anti-angiogenic factors in the pathogenesis of PE has gained the most attention in the past decade.

Vascular endothelial growth factor (VEGF), a member of VEGF family, is a major angiogenic factor and potential regulator of endothelial cell proliferation [5]. During pregnancy, VEGF is essential for the proliferation of trophoblasts, the development of embryonic vasculature, and the growth of maternal and foetal blood cells in utero [6]. The human VEGF gene is located on chromosome 6p21.3 and consists of 8 exons with alternate splicing, forming a family of proteins. Several studies have been performed to elucidate its significance in PE. Emerging evidence suggested that VEGF is elevated in PE and correlates with the severity of disease [7]–[10]. Although sources have found depressed levels of VEGF in the sera of PE women [11]–[14], these results can be explained by the fact that quantification of VEGF in pregnancy is affected by interference from binding proteins [15]. The immediate importance of VEGF lies in its implications for the early identification of PE. Bosio PM, et al. [16] performed a longitudinal study, which VEGF levels were measured serially from 10 weeks' gestation, and found VEGF levels were elevated from 28 weeks onwards in women who subsequently developed PE. Similar with Bosio’s results, Hunter A, et al. [17] also performed a similar prospective study and found that VEGF levels in women with PE were significantly different from normotensive women beginning at 30 weeks' gestation. These data suggest the potential role of VEGF in the pathogenesis of PE, and abnormal VEGF levels in pregnancy before the clinical onset of PE may be useful as a predictive marker.

There are several common single nucleotide polymorphisms (SNPs) in the VEGF gene, including +936 (rs3025039), −634 (rs2010963), −1154 (rs1570360), and −2578 (rs699947) positions, which could alter gene expression and protein production, and alter the risk of developing diseases characterized by deranged angiogenesis [18]–[20]. Over the past decade, considerable epidemiological studies have focused on associations between VEGF polymorphisms and PE susceptibility. However, the results remain controversial or inconclusive. To address this issue, we performed an updated systemic review and a meta-analysis of all eligible case-control studies to provide insights into the associations between VEGF polymorphisms and susceptibility to PE, which may promote our understanding of the exact role of VEGF gene in the etiology of PE and early identification of the patients at risk of PE.

## Materials and Methods

### Search Strategy

A systematic literature search of MEDLINE (updated to June, 2013), Embase (updated to June, 2013), Web of Science (updated to June, 2013), and CNKI (Chinese National Knowledge Infrastructure) databases was conducted by two study investigators (D.C. and Y. H.) independently for all relevant articles. Key words used in the research included “vascular endothelial growth factor”, “VEGF”, “preeclampsia”, “polymorphism”, “*single-nucleotide polymorphism*”, “variant”, “genotype”, “mutation”, “pregnancy induced hypertension”, and “gestational hypertension”.

### Inclusion and Exclusion Criteria

Studies eligible for inclusion in this meta-analysis should meet the following criteria: (a) case-control studies or cohort studies focused on associations between VEGF polymorphism and risk of PE; (b) patients have clinically confirmed PE; (c) The studies provided the number of cases and controls for various genotypes. PE was defined as elevated blood pressure (≥140/90 mm Hg on two measurements ≥6 hours apart) with ≥1+ proteinuria or 300 mg/24 hours after the 20th week of pregnancy [21]. The exclusion criteria of the meta-analysis were: (a) animal studies; (b) meta-analyses, letters, reviews, meeting abstracts, or editorial comments; (c) studies with duplicate data or incomplete data. When an individual author published several articles obtained from the same patient population, only the newest or most complete article was included in the analysis.

### Data extraction

Information was carefully extracted from all the eligible publications. The following data were collected from each study: first author’s name, publication date, country, ethnicity, source of controls, genotyping method, total numbers of cases and controls, and number of cases and controls for each VEGF polymorphism. An attempt was made to contact authors if data presentation was incomplete or if it was necessary to resolve an apparent conflict or inconsistency in the article. Any disagreements were resolved by consensus.

### Statistical Analysis

Review manager 5.1 program provided by the Cochrane Library and Stata (Version12.0, Stata Corporation) were used to perform all the statistical analysis. The association was evaluated with the use of the allelic contrast (mutation [M] allele versus wild [W] allele), as well as the dominant model (WM+MM versus WW), the recessive model (MM versus WM+WW), and the homozygous contrast [MM versus WW], respectively. Two models of pooling data for dichotomous outcomes were conducted: the random-effects model and the fixed-effects model. The pooled statistical analysis was calculated using the fixed effects model, but a random-effect model was performed when the *P* value of heterogeneity test was <0.1. The OR and 95% CI were calculated for each study, and the combined OR and 95% CI were calculated for all eligible studies. The significance of the combined OR was determined by the Z-test, in which *P*<0.05 was considered significant. Heterogeneity assumption was assessed by the Chi-square based Q test and was regarded to be statistically significant if *P*<0.10. The potential publication bias was assessed by Begg’s funnel plot and Egger’s test [22], [23].

## Results

### The Characteristics of Included Studies

**Figure 1:**
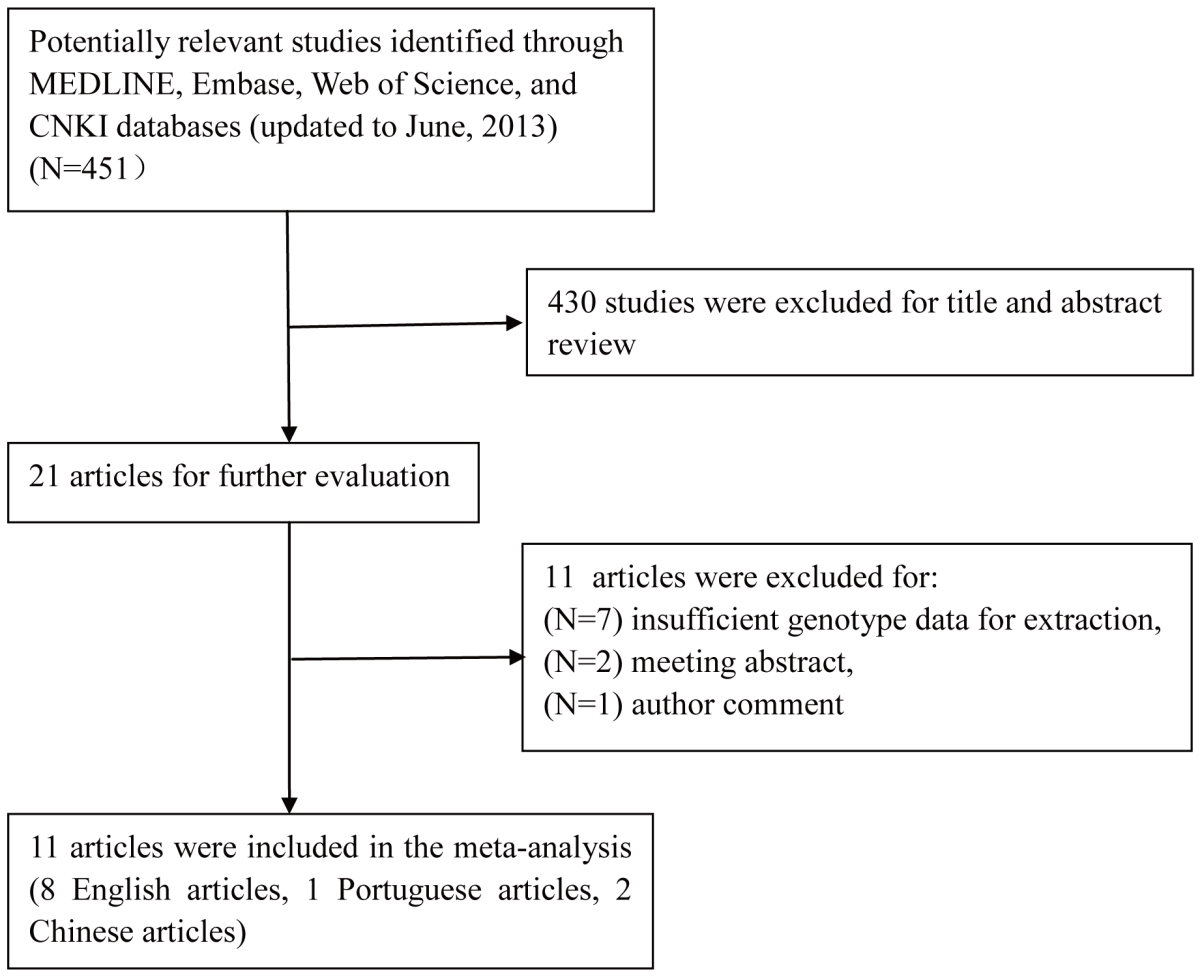
Flow chart of study selection. (CNKI, Chinese National Knowledge Infrastructure).

The flow chart that displays the study selection process was shown in Figure 1. Briefly, a total of 451 results were identified after an initial search from the Pubmed, Embase, Web of science, and CNKI databases. After screening the titles or abstracts, 430 publications were excluded because of case reports, or reviews, or non-relevant research. According to the inclusion criteria, 11 case-control studies were finally included in this meta-analysis [18], [20], [24]–[32]. A total of 1,069 PE cases and 1,315 controls were included in this study.

The characteristics of 11 included studies were summarized in Table 1. There are eight case-control studies concerning +936C/T polymorphism [18], [20], [24], [26], [27], [29], [31], [32], seven case-control studies concerning −2578C/A polymorphism [18], [20], [24], [25], [28]–[30], five case-control studies concerning −634G/C polymorphism [20], [26], [29], [30], [32], three case-control studies concerning −1154G/A polymorphism [20], [25], [30]. Controls were selected from healthy pregnant population in all the studies. The genotype distributions among the controls of all studies were in agreement with Hardy–Weinberg equilibrium (HWE) except for one study for the −634G/C.

**Table 1:** Baseline characteristics of the 11 eligible studies for the analysis of VEGF polymorphism.

Studies	Year	Country	Ethnicity	Source of controls	Sample size	SNP studied	Genotyping method	HWE
Shim JY	2007	Korea	Asian	HB	110/209	+936C/T	PCR-RFLP	0.686
Kim YJ	2007	Korea	Asian	HB	223/237	+936C/T, −634G/C	SNaPShot assay	0.340, 0.01
Cunha VM	2010	Brazil	Latinos	HB	52/28	+936C/T, −2578C/A	PCR-RFLP	0.331,0.160
Chedraui P	2013	Ecuador	Latinos	HB	31/31	+936C/T, −2578C/A, −634G/C, −1154G/A	Sequencing	0.952, 0.479, 0.952, 0.797
Andraweere PH	2013	Australia	Caucasians	HB	174/168	+936C/T, −2578C/A,	MassARRAY system.	0.748, 0.440
He Y	2011	China	Asian	HB	61/43	+936C/T, −634G/C	Sequencing	0.550, 0.361
Huang Y	2009	China	Asian	HB	128/231	+936C/T,	PCR-RFLP	0.07
Garza-Veloz I	2011	Mexico	Latinos	HB	86/78	−2578C/A, −1154G/A	PCR-RFLP	0.865, 0.442
Bányász I	2006	Hungary	Caucasians	HB	84/96	−2578C/A	PCR-RFLP	0.180
Sandrim VC	2008	Brazil	Latinos	HB	94/108	−2578C/A, −1154G/A, −634G/C	Taqman AllelicDiscrimination	0.210, 0.142,0.341
Papazoglou D	2004	Sweden	Caucasians	HB	42/73	+936C/T, −2578C/A, −634G/C	PCR-RFLP	0.906, 0.131,0.251

HB, hospital-based controls. HWE, Hardy–Weinberg equilibrium.

### Quantitative Synthesis

#### VEGF +936C/T

**Figure 2:**
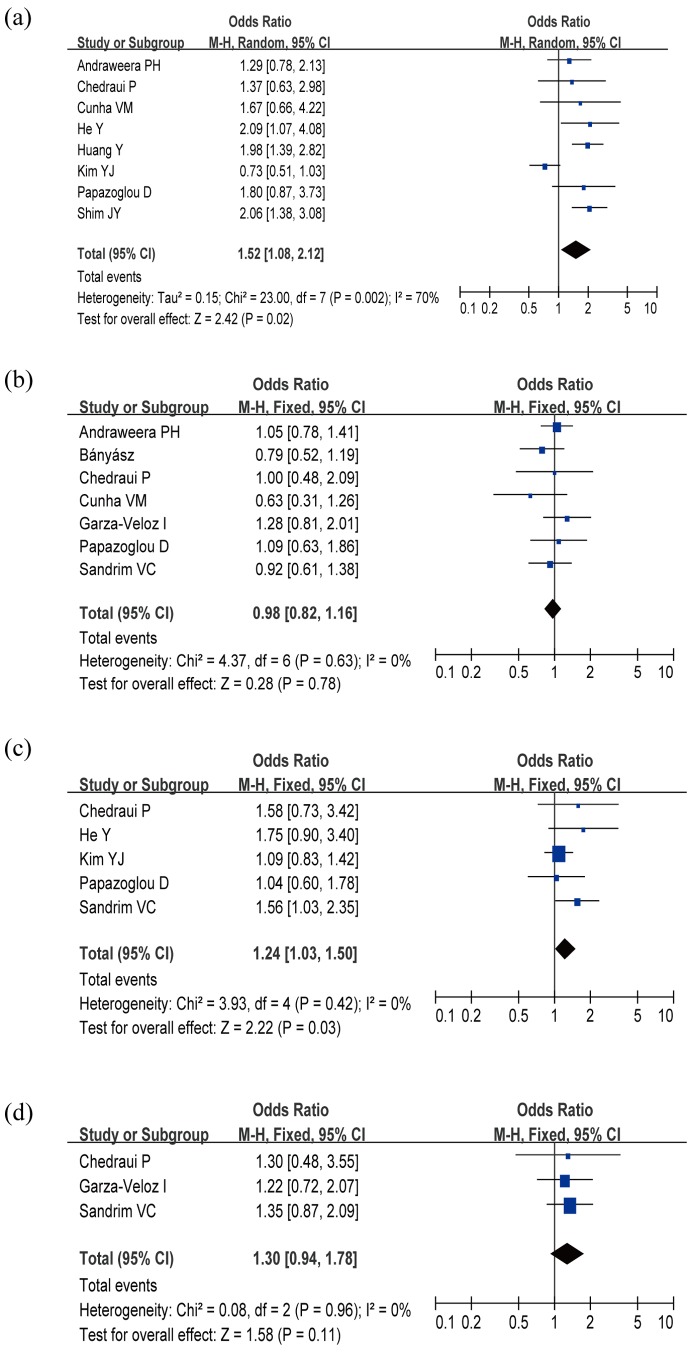
Overall ORs for the association between VEGF gene polymorphisms and the risk of preeclampsia under allele contrast. (a) VEGF +936C/T; (b) VEGF–2578C/A; (c) VEGF –634G/C; (d) VEGF –1154G/A.

The evaluation of association between VEGF polymorphisms and PE risk was presented in Table 2. As for VEGF +936C/T polymorphism, 805 PE cases and 1,033 controls were included in the meta-analysis. Heterogeneity obviously existed under allele contrast and dominant models (all *P*<0.1), which might be the result of the difference in ethnicity, country, source of controls, and genotype methods, so random effects model was conducted to pool the results. Overall, the significant association was found between VEGF +936C/T polymorphism and risk of PE in allele contrast (OR = 1.52, 95%CI = 1.08–2.12, *P* = 0.02, Figure 2a), dominant model (OR = 1.59, 95%CI = 1.06–2.37, *P* = 0.02), recessive model (OR = 1.86, 95%CI = 1.17–2.96, *P* = 0.009), and homozygous contrast (OR = 2.07, 95%CI = 1.29–3.31, *P* = 0.002). The results suggested that the T allele carriers might have an increased risk of PE compared with those individuals with the CC homozygote.

**Table 2:** Main results for the VEGF polymorphism with the risk of PE based on OR and 95% CI.

Genotype comparison	OR [95% CI]	Z (*P* value)	Heterogeneity of study design		Model
			χ^2^	I^2^	
**VEGF +936C/T (805 cases, 1033 controls)**					
T vs. C	1.52 [1.08, 2.12]	2.42 (0.02)	23.00	70%	Random
TT+CT vs. CC (dominate model)	1.59 [1.06, 2.37]	2.26 (0.02)	23.14	70%	Random
TT vs. CT+CC (recessive model)	1.86 [1.17, 2.96]	2.63 (0.009)	6.30	0%	Fixed
TT vs. CC (homozygous contrast)	2.07 [1.29, 3.31]	3.03 (0.002)	9.06	23%	Fixed
**VEGF** –**2587C/A (572 cases, 574 controls)**					
A vs. C	0.98 [0.82, 1.16]	0.28 (0.78)	4.37	0%	Fixed
AA+CA vs. CC (dominant model)	1.00 [0.78, 1.29]	0.02 (0.98)	8.29	28%	Fixed
AA vs. CA+ CC(recessive model)	0.93 [0.68, 1.26]	0.47 (0.64)	3.62	0%	Fixed
AA vs. CC (homozygous contrast)	0.93 [0.65, 1.32]	0.42 (0.68)	2.46	0%	Fixed
**VEGF**–**634G/C (441 cases, 485 controls)**					
C vs. G	1.24 [1.03, 1.50]	2.22 (0.03)	3.93	0%	Fixed
CC+GC vs. GG (dominant model)	1.42 [1.09, 1.86]	2.56 (0.01)	2.11	0%	Fixed
CC vs. GC+GG (recessive model)	1.20 [0.78, 1.85]	0.84 (0.40)	4.66	14%	Fixed
CC vs. GG (homozygous contrast)	1.38 [0.93, 2.04]	1.61 (0.11)	4.17	4%	Fixed
**VEGF** –**1154G/A (217 cases, 209 controls)**					
A vs. G	1.30 [0.94, 1.78]	1.58 (0.11)	0.08	0%	Fixed
AA+GA vs. GG (dominant model)	1.25 [0.82, 1.88]	1.04 (0.30)	0.36	0%	Fixed
AA vs. GA+GG (recessive model)	1.63 [0.87, 3.07]	1.51 (0.13)	0.55	0%	Fixed
AA vs. GG (homozygous contrast)	1.66 [0.87, 3.17]	1.55 (0.12)	0.61	0%	Fixed

OR, odds ratio.

#### VEGF –2578C/A

A total of 572 cases and 574 controls from 7 case-control studies were included for data synthesis. The meta-analysis results showed that VEGF –2578C/A polymorphism was not linked to the risk of PE under all genetic models (allele contrast, OR = 0.98, 95%CI = 0.82–1.16, *P* = 0.78, Figure 2b; dominant model, OR = 1.00, 95%CI = 0.78–1.29, *P* = 0.98; recessive model, OR = 0.93, 95%CI = 0.68–1.26, *P* = 0.64; and homozygous contrast, OR = 0.93, 95%CI = 0.65–1.32, *P* = 0.68).

#### VEGF –634G/C

A total of 441 cases and 485 controls from five case-control studies were included for data synthesis. We noted a statistically significant finding in the pooled analysis for the VEGF –634G/C in PE. In the allelic contrast, a significantly increased risk was presented for the comparison of the C allele with the G allele (OR = 1.24, 95% CI = 1.03–1.50, *P* = 0.03, Figure 2c). There was also significance in the dominant model (OR = 1.42, 95%CI = 1.09–1.86, *P* = 0.01), while the differences in recessive model (OR = 1.20, 95%CI = 0.78–1.85, *P* = 0.40) and homozygous contrast (OR = 1.38, 95%CI = 0.93–2.04, *P* = 0.11) were not significant.

#### VEGF –1154G/A

A total of 217 cases and 209 controls from only three case-control studies were included for data synthesis. The meta-analysis results showed that VEGF –1154G/A polymorphism was not associated with the risk of PE under all genetic models (allele contrast, OR = 1.30, 95%CI = 0.94–1.78, *P* = 0.11, Figure 2d; dominant model, OR = 1.25, 95%CI = 0.82–1.88, *P* = 0.30; recessive model, OR = 1.63, 95%CI = 0.87–3.07, *P* = 0.13; and homozygous contrast, OR = 1.66, 95%CI = 0.87–3.17, *P* = 0.12).

### Sensitivity Analysis

We carried out a sensitivity analysis by removing one single study each time. The results indicated no significant differences before and after the removal for pooled analysis, suggesting high stability of our results.

### Publication Bias

**Figure 3:**
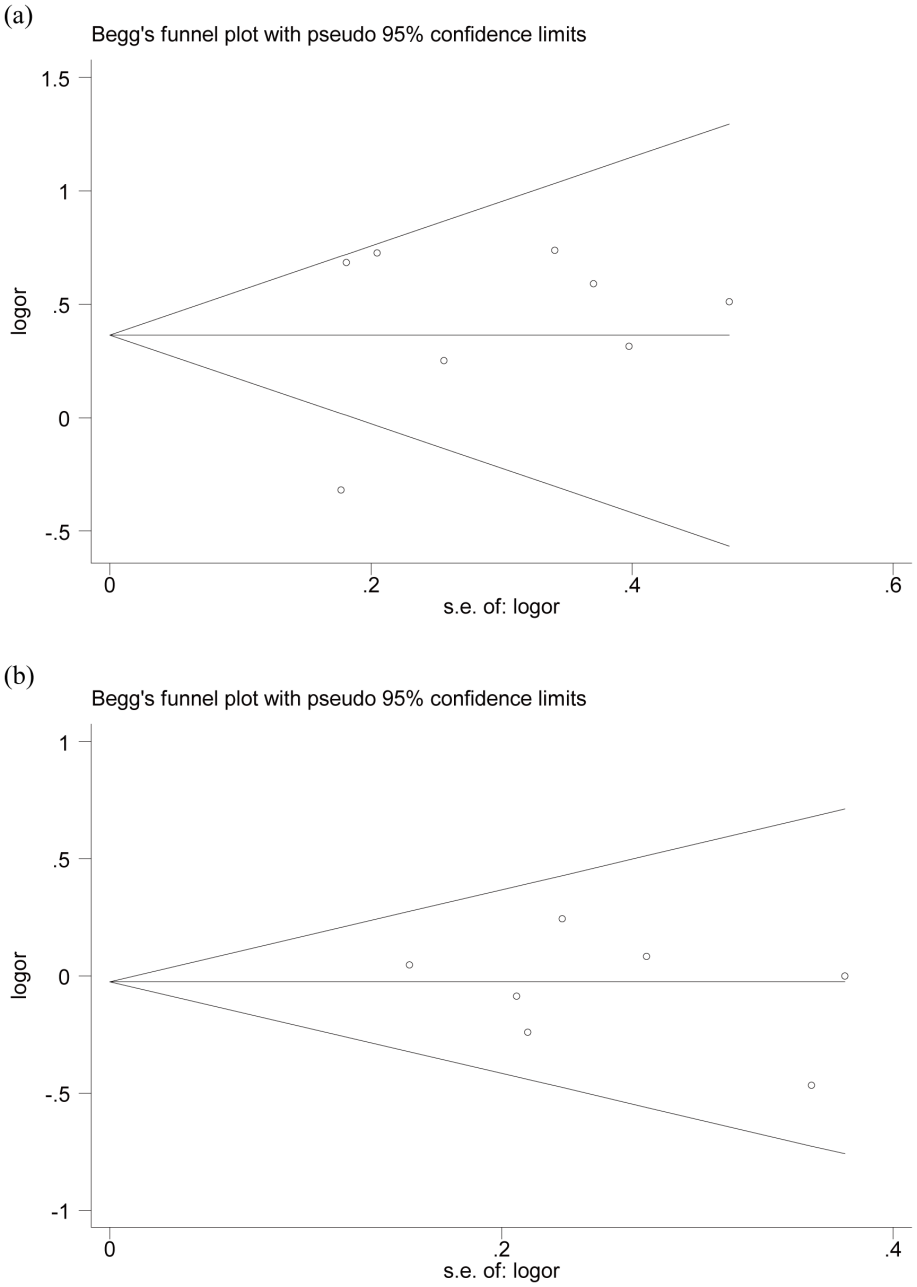
Begger’s funnel plot of the meta-analysis of VEGF +936C/T and–2578C/A polymorphism with preeclampsia under allele contrast. Each point represents a separate study for the indicated association. Log[OR], natural logarithm of OR. Horizontal line, mean magnitude of the effect. Note: Funnel plot with pseudo 95% confidence limits was used.

Begg’s funnel plot and Egger’s test were performed to assess the publication bias of included studies. The shapes of the funnel plots did not reveal any evidence of obvious asymmetry under the allele contrast (+936 C/T, *P* = 0.711; –2578C/A, *P* = 0.548) (Fig. 3). Egger’s test also did not show any significantly statistical evidence of publication bias under the allele contrast (+936C/T, *P* = 0.556; –2578C/A, *P* = 0.508), which indicated low risk of publication bias in this meta-analysis.

## Discussion

The cause of PE remains largely unknown but there is growing evidence that an imbalance between VEGF family and possible anti-angiogenic factors is closely related to the pathogenesis of PE. During a normal healthy pregnancy, a highly coordinated program ensures that the placenta and fetus are supplied with an adequate blood supply necessary for proper oxygen and nutrient delivery. In the development of PE, defective angiogenesis and the related fetoplacental vascular dysfunction are therefore considered key steps. Therefore, one area of intensive research in recent years focused on VEGF signaling pathway in PE development, which is necessary for the maintenance of proper endothelial cell function and health.

Recently, case-control studies have shown associations between VEGF gene polymorphisms and PE. In the present study, the associations between four genetic polymorphisms in VEGF genes and PE were investigated from eleven eligible case-control studies. We found two SNPs were statistically associated with PE, which suggests that genetic variation in +936C/T and –634 G/C loci might play a role in this disease. To our knowledge, this is the first meta-analysis to evaluate the relationship between SNPs in the VEGF gene and the risk of PE.

VEGF is a multifunctional cytokine that plays a pivotal role in angiogenesis in vivo. There are several polymorphisms in the VEGF gene and many polymorphisms are associated with the protein production. Among these, four VEGF SNPs, +936C/T in the 3′-untranslated region, –634G/C in the 5′-untranslated region, and –2578C/A and –1154G/A in the promoter region, were reported to modulate VEGF expression [33]–[35]. In our analysis, we found significant correlation between the +936C/T polymorphism and the risk of PE. Subjects carrying the T allele have significantly higher risk of PE than subjects carrying the VEGF +936CC genotype. In accordance with the result of Renner W, *et al*., VEGF +936C/T variant may be an important determinant of VEGF plasma levels. Livingston JC, *et al*. [36] reported that patients with PE had decreased maternal serum concentrations of VEGF, and Madazli R, *et al*, [37] also suggested that the clinical severity of PE seems to correlate with the severity of the cytokine abnormalities including VEGF. As for VEGF –634G/C, we found VEGF –634G/C polymorphism was associated with the risk of PE under allele contrast and dominant model. In searching for possible factors that might have impacted the results, some clinical factors, such as ethnicity, genotype method, different gestational weeks, and HWE test might lead to bias. Taking into account that VEGF polymorphisms has an important role during pregnancy outcome, the present data strongly suggest that a reduced ability of mother's tissue to up-regulate VEGF (as predicted by the 936C/T VEGF or –634 G/C polymorphism) has a considerable effect on PE. In addition, our meta-analysis suggested that VEGF –2578C/A and –1154G/A polymorphisms had no association with PE risk in all examined patients. Considering the included case-control studies for both polymorphisms are relatively small (5 studies for –634G/C polymorphisms, and only 3 studies for –1154G/A), larger number of relevant studies is needed in future to validate these results.

Several limitation of this present study should be acknowledged. Although an adequate search strategy was used to identify eligible studies, it was possible that some eligible studies were not included, which may have biased our results. In addition, we analyzed a relatively small number of studies (11 studies), including 1,069 cases, which may reduce the statistical power for identifying possible associations between the VEGF polymorphisms and PE risk. Moreover, other clinical factors such as age, ethics, and different gestational weeks in each study might lead to bias. Determining whether or not these factors influence the results of this meta-analysis would need further investigation. Therefore, a more well-designed study with larger sample sizes is needed to further assess the precise effect of these VEGF polymorphisms in PE, and further studies are needed to see if other genotype of VEGF confers a risk of PE in the populations.

### Conclusion

Our meta-analysis suggested that VEGF –2578C/A or –1154G/A polymorphism had no association with PE risk in all examined patients, whereas there was an association between VEGF +936C/T or –634G/C polymorphism and risk of PE. However, further studies are necessary in order to warrant and validate the associations between VEGF gene polymorphisms and PE risk.

## Supporting Information

Figure S1 **PRISMA 2009 Flow Diagram.**
(DOC)

Checklist S1 **PRISMA 2009 Checklist.**
(DOC)
