# Supplementary material for: Vascular Endothelial Growth Factor +936C/T, –634G/C, –2578C/A, and –1154G/A Polymorphisms with Risk of Preeclampsia: A Meta-Analysis
Source: PLoS One. 2013 Nov 4;8(11):e78173. doi: 10.1371/journal.pone.0078173 (PMC3817179; doi:10.1371/journal.pone.0078173)
Supplement: Figure S1 — PRISMA 2009 Flow Diagram. (DOC) [file pone.0078173.s001.doc]

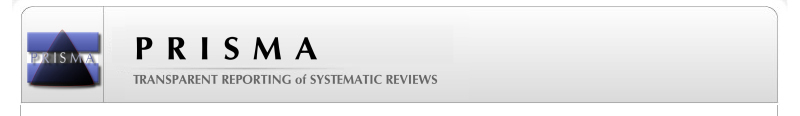
**PRISMA 2009 Flow Diagram**

**Screening**

**Included**

**Eligibility**

**Identification**

Records identified through database searching
(n = 451 )

Additional records identified through other sources
(n =0 )

Records after duplicates removed
(n =0 )

Records screened
(n =451 )

Records excluded
(n = 431 )

Full-text articles assessed for eligibility
(n =21 )

Full-text articles excluded, with reasons
(n = 10 )

Studies included in qualitative synthesis
(n =11 )

Studies included in quantitative synthesis (meta-analysis)
(n =11 )
